# Reply to: Deep reinforced learning heuristic tested on spin-glass ground states: The larger picture

**DOI:** 10.1038/s41467-023-41108-w

**Published:** 2023-09-14

**Authors:** Changjun Fan, Mutian Shen, Zohar Nussinov, Zhong Liu, Yizhou Sun, Yang-Yu Liu

**Affiliations:** 1 College of Systems Engineering, National University of Defense Technology, Changsha, 410073 China; 2 Department of Physics, Washington University in St. Louis, Campus Box 1105, 1 Brookings Drive, St. Louis, MO 63130 USA; 3 Rudolf Peierls Centre for Theoretical Physics, University of Oxford, Oxford, OX1 3PU UK; 4 Department of Computer Science, University of California, Los Angeles, CA 90024 USA; 5 Channing Division of Network Medicine, Department of Medicine, Brigham and Women’s Hospital and Harvard Medical School, Boston, MA 02115 USA; 6 Center for Artificial Intelligence and Modeling, The Carl R. Woese Institute for Genomic Biology, University of Illinois at Urbana-Champaign, Champaign, IL 61820 USA

**Keywords:** Mathematics and computing, Statistical physics

**replying to** S. Boettcher *Nature Communications* 10.1038/s41467-023-41106-y (2023)

Here we provide a comprehensive response to the Comment written by Stefan Boettcher. We argue that the Comment did not account for the fairness of the comparison between different methods in searching for the spin-glass ground states. We demonstrate that, with a reasonably larger number of initial spin configurations, our results agree with the asymptotic scaling form assumed by finite-size corrections.

## 3D Edwards-Anderson (EA) model

**Fig. 1:**
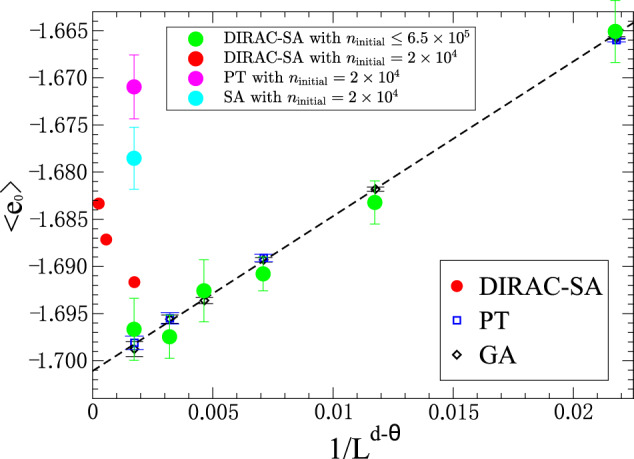
With a reasonably large *n*_initial_, our DIRAC-SA results agree well with larger picture suggested by FSC. FSC assumes that the average ground state energy per spin of a given *d*-dimensional EA system of size *N* = *L*^*d*^ has the form ${ < {e}_{0} > }_{N}={ < {e}_{0} > }_{N=\infty }+Ax+\cdots \,$, where *x* = 1/*L*^*d*−*θ*^ and *d* − *θ* ≈ 2.76. Ignoring the higher order terms, this form is shown as the dashed line here. The red, magenta and cyan points are ${ < {e}_{0} > }_{N}$ for *N* = 10^3^ computed by DIRAC-SA, PT, and SA, respectively, all with *n*_initial_ = 2.0 × 10^4^. The green points represent ${ < {e}_{0} > }_{N}$ for *N* = 4^3^, 5^3^, 6^3^, 7^3^, 8^3^, 10^3^, with *n* = 850, 900, 820, 120, 221, 50 instances respectively, computed by DIRAC-SA with *n*_initial_ ≤ 6.5 × 10^5^. Adapted from Figure 1 of Boettcher, S., Nat Commun. (submitted)^11^.

In Fig. 5 of our paper^1^, we plotted the disorder-averaged energy per spin (denote as *e*_0_) as a function of the number of initial spin configurations (denoted as *n*_initial_) for different methods to benchmark those methods on large 3D EA Ising spin glass instances with Gaussian disorder. The Comment pointed out that DIRAC-SA (a variant of our DIRAC method) did not reach the ground states for those systems, as indicated by the large deviation of the three red points from the asymptotic scaling form assumed by finite-size corrections (FSC), see Fig. 1 of the Comment and this response letter. However, as we explicitly mentioned in the caption of Fig. 5 in our paper^1^, we only ran all the tested algorithms up to a small *n*_initial_ = 2.0 × 10^4^, which is much smaller than the number required to reach the ground state, as reported in the literature. For instance, Ref. ^2^ reported that, to reach the ground state for 3*D*, *L* = 10 systems, the parallel tempering (PT) method requires *n*_initial_ = 3.2 × 10^7^, which is 1600 times larger than the number of initial spin configurations we used. Such a big difference in terms of *n*_initial_ is certainly not inconsequential. We did not expect any of the methods to reach the ground state with *n*_initial_ = 2.0 × 10^4^ for large 3D EA instances with Gaussian disorder. Indeed, for 3*D*, *L* = 10 systems, with *n*_initial_ = 2.0 × 10^4^, PT and simulated annealing (SA) did not reach the expected ground state either (see the magenta and cyan points in Fig. 1 of this response). In fact, with the same *n*_initial_, results of these two methods are even farther away from the FSC line than DIRAC-SA for 3*D*, *L* = 10 systems (see the third red point in Fig. 1 of this response). Without specifying the number of initial spin configurations, we think it is unfair and meaningless to compare different methods in searching for the ground states of large spin-glass instances.

In our paper^1^ we did not try a larger *n*_initial_ for two reasons. First, we had already demonstrated the ability of DIRAC to reach the exact ground states for small systems (which can be confirmed by the branch-and-bound-based solver Gurobi), as shown in Fig. 4 of our paper^1^. Second, we did not find it necessary to invest extensive computational resources in an “arms race” fashion of computing the “ground states” of these large systems for which exact solvers cannot confirm the results. Also, to achieve the (true) ground states the required *n*_initial_ may be exponential in the system size. There is no exception for DIRAC or any other heuristic methods. Our paper aimed to demonstrate the effectiveness and efficiency of DIRAC over other methods at the same *n*_initial_, rather than to confirm the asymptotic scaling form assumed by FSC. We appreciate the “larger picture” mentioned in the Comment. But it was beyond the scope of our paper.

Since the Comment questioned the ability of our method to reach the ground state for large systems, we think it is necessary to perform heavier computations with a larger *n*_initial_ to directly address the Comment. For 3*D*, * L* = 10 systems with *n* = 50 instances, we found that, with *n*_initial_ = 6.5 × 10^5^, about 2% as that needed for PT, the average energy per spin computed by DIRAC-SA could indeed reach the asymptotic scaling form assumed by FSC (see the leftmost green point in Fig. 1). We also plotted *e*_0_ computed by DIRAC-SA for 3*D*, *L* = 4, 5, 6, 7, 8, with *n* = 850, 900, 820, 120, 221 instances respectively, in the same figure. We found that they agree well with the FSC line. These results clearly demonstrate that the importance of using a large *n*_initial_ to achieve results consistent with the prediction of FSC. We are grateful that the Comment helped us clarify this point. As mentioned above, confirming the asymptotic scaling form assumed by FSC was not the original goal of our paper.

## Sherrington-Kirkpatrick (SK) model

Fig. 2 of the Comment acknowledged that our results for the SK model are consistent with the asymptotic scaling form assumed by FSC, although in the figure we could still see a deviation from the FSC line for SK model of *N* = 64. We believe this deviation is simply due to the small number of instances (*n* = 50) used in our calculation. We notice that with *n* = 50 instances the results offered by the extremal optimization (EO) heuristic also deviate from the FSC line, especially for *N* = 125. We argue that DIRAC needs more instances to reach the FSC line, just like the EO case. After all, only the average over many different instances may be expected to behave as a smooth function of *N*^3^.

The Comment also pointed out that the system sizes we considered are relatively small. We emphasize that, as a reinforcement-learning framework based on graph neural network, DIRAC was not specifically designed for SK models with a complete graph topology. We believe that, to compute ground states for larger SK instances, DIRAC would have to be modified to explicitly consider the complete graph topology. However, this was beyond the scope of our paper.

## Competitive methods

It is a pity that in our paper we did not explicitly cite any papers on the genetic algorithm^3,4^ (GA) or extremal optimization (EO) heuristic^5–7^. We did cite a book^8^ on the use of those heuristic methods for computing the spin-glass ground state though, as also pointed out by the Comment. In our paper, we did not compare the performance of DIRAC with that of GA and EO either. This is mainly because PT and GA were commonly used to compute the ground state of the EA Ising spin glass model with Gaussian disorder^2,9^, and Ref. ^9^ reported that a simple PT algorithm performs as well as GA found in the literature. Hence, we chose PT as a competitive method of DIRAC. We did consider two classical heuristic methods: SA and Greedy algorithm. Overall, we think comparing DIRAC with those methods is sufficient to demonstrate its superiority.

## Running time

In our main text, we primarily focused on comparing the value of *n*_initial_ among different methods. We believe this is a fair comparison since this metric remains unaffected by the computational environment, programming language, or system load during testing. It can also be interpreted as the number of ‘exploration steps’ taken by each algorithm, which, to some extent, reflects the algorithm’s level of ‘intelligence’. As an extreme example, Fig. 7 in our main text demonstrates that even a simple DIRAC^1^ method can achieve the ground state of an anti-ferromagnetic model with the theoretically minimal number of exploration steps.

Nevertheless, we understand that some readers may inquire about the actual running time or ‘wall clock time’ of our algorithm. Therefore, we have provided two tables, Tables 1 and 2, which present the typical running times of DIRAC and SA on a laptop equipped with an Intel(R) Core(TM) i5-10400 processor and Nvidia(R) Geforce(R) RTX 2070 graphics card, and also a server equipped with an Intel(R) Xeon(R) Gold 6278C processor and Nvidia(R) Tesla(R) V100 graphic card. The running times of other algorithms, such as DIRAC-SA, DIRAC-PT or PT, can be roughly estimated based on these values. For instance, for *n*_initial_ = 5000, the time cost of DIRAC-SA is roughly the sum of 2500 DIRAC^1^ and 2500 SA sweeps. Also, it is expected that the time required for an SA sweep and a PT sweep would not exhibit a significant difference.

**Table 1:** Average running time for *n*_initial_ = 1 on Intel(R) Core(TM) i5-10400 @2.9GHz and Nvidia(R) Geforce(R) RTX 2070

	3*D*, *L* = 10	3*D*, *L* = 20	3*D*, *L* = 30
DIRAC^1^(Python,C++;GPU)	~2.7s	~47s	~320s
DIRAC^1^(MATLAB;GPU)	~1.2s	~4.8s	~14s
SA sweep(Python,C++)	~0.014s	~0.13s	~0.45s
SA sweep(MATLAB)	~0.020s	~0.18s	~0.77s
SA sweep(C++)	~0.0024s	~0.03s	~0.11s

This table (and also Table 2) presents the typical running times, measured in seconds, for various system sizes, different methods, and different implementations

**Table 2:** Average running time for *n*_initial_ = 1 on Intel(R) Xeon(R) Gold 6278C CPU @ 2.60GHz and Nvidia(R) Tesla(R) V100

	3*D*, *L* = 10	3*D*, *L* = 20	3*D*, *L* = 30
DIRAC^1^(Python,C++;GPU)	~2.8s	~49s	~330s
DIRAC^1^(MATLAB;GPU)	~0.5s	~1.1s	~3.2s
SA sweep(Python,C++)	~0.015s	~0.18s	~0.61s
SA sweep(MATLAB)	~0.01s	~0.09s	~0.41s
SA sweep(C++)	~0.003s	~0.034s	~0.13s

We acknowledge that our DIRAC code was not optimized for achieving the shortest running time. However, even in such case, in terms of the running time taken to reach the same energy, DIRAC’s running time is not at a disadvantage, if not in an advantageous position. For example, a comparative test was conducted on the same 3*D*, *L* = 10 systems for SA and DIRAC-SA. An average energy density of approximately −1.6956 can be achieved with 10^4^ SA (with *n*_initial_ = 5 × 10^7^), while reaching the same energy level only requires 47 DIRAC-SA (with *n*_initial_ = 2.35 × 10^5^). Even after taking into account the running time differences between DIRAC^1^ and SA sweep shown in Table 2, we can estimate that the MATLAB version of DIRAC-SA is still ~2.5 times faster than the C++ version of SA. Despite the additional use of GPU, we believe that compared to SA, DIRAC can more naturally benefit from GPU acceleration, as the time consumption of DIRAC is primarily on matrix multiplication.

The running time of DIRAC is influenced by many factors, so there may still be room for improvement. In fact, during the development of DIRAC, we discovered a significant time overhead due to communication between C++ modules, the Tensorflow session, and the Python code. (As an indirect evidence, it can be observed that for this code, there is no significant difference in the running time between the RTX 2070 and V100 GPU). Hence, employing a unified programming language could greatly improve performance, as demonstrated by the MATLAB running times listed in Tables 1 and 2. In addition to these findings, we have identified several other ways to accelerate the code:

- Implement the code in an incremental way. For instance, in the context of SA, when attempting to flip a spin, it is sufficient to compute the energy of that specific spin. However, in the current version of the DIRAC code, whenever the spin configuration is altered, all the Q values need to be recomputed, which is clearly not efficient. To improve this, we can modify the code to update only the affected Q values when a spin is flipped, rather than recomputing all of them. This incremental approach will optimize the computation process.
- Matrix chain multiplication. In the current version of the DIRAC code, we did not optimize the order of the matrix multiplication. This could also possibly be a way to optimize the computation running time.
- Programming language. We believe that if the entire code is written in C++/CUDA, the running time should be further reduced.

On the other hand, for the DIRAC^1^ code written in MATLAB, the performance difference of GPUs is still very noticeable, compared to the insignificant differences in single-core performance among modern CPUs; for instance, see the SA sweep running time on different machines. For instance, when we replaced the RTX 2070 with the V100 server GPU, the running time was reduced by nearly 2–4 times. Furthermore, from the table, we can observe that for the DIRAC^1^ code written in MATLAB, its time complexity appears to be even less than linear. This may suggest that the performance of GPUs is not fully utilized, at least in smaller systems. In general, we believe that DIRAC has significant potential for further development in terms of computational time.

## Methods

The hyperparameters used in the DIRAC-SA algorithm mentioned in this paper are the same as the default hyperparameters in the GitHub code^1^. In addition, the MATLAB version of DIRAC^1^ that we used for the running time test has also been updated on GitHub^1^. The details of the computing environments have been provided in the section “Running Time”.

## Data Availability

The data used to reproduce the results in this paper are publicly available^10^.

## Code availability

The source code of DIRAC (and its variants), as well as the two baseline methods, SA and PT, are publicly available^10^ or on GitHub (https://github.com/FFrankyy/DIRAC.git).
